# The Particle Size Effect: Cytotoxicity and Cellular Uptake of Polystyrene Nanoplastics in Human Keratinocytes

**DOI:** 10.3390/toxics14060507

**Published:** 2026-06-10

**Authors:** Xiaofeng Bai, Fan Wu, Yi Qin, Qitian Fu, Jun Wang, Yao Pan

**Affiliations:** 1 Department of Cosmetics, School of Light Industry Science and Engineering, Beijing Technology and Business University, Beijing 100048, China; 2431041306@st.btbu.edu.cn (X.B.); 2230401031@st.btbu.edu.cn (F.W.); 2330402109@st.btbu.edu.cn (Y.Q.); 2431042188@st.btbu.edu.cn (Q.F.); 2330402112@st.btbu.edu.cn (J.W.); 2 Beijing Key Laboratory of Plant Research and Development, Beijing 100048, China; 3 Academy for Interdisciplinary Studies, Beijing Technology and Business University, Beijing 100048, China; 4 National Medical Products Administration Cosmetics Innovation and Research Base, Beijing 100048, China

**Keywords:** polystyrene nanoplastics, skin toxicity, oxidative stress, autophagy, particle size effect

## Abstract

Nanoplastics from plastic waste degradation pose a growing environmental health risk, yet size-dependent dermal effects remain poorly understood. This study investigated polystyrene nanoplastics of 50, 100, and 200 nm using ex vivo porcine skin and in vitro human keratinocyte models. Skin permeation, cellular uptake, viability, oxidative stress, inflammation, autophagy, and transcriptomic pathways were assessed. Enhanced nanoparticle penetration was observed in barrier-disrupted skin, primarily via hair follicles, with smaller particles showing greater intracellular accumulation. Transcriptomics revealed disruptions in oxidative stress, inflammation, endocytosis, and autophagy pathways. Specifically, 50 nm particles induced the strongest oxidative stress via Nrf2 activation and triggered sustained autophagy, leading to proliferation inhibition and time-dependent inflammation. In contrast, 100 nm particles caused moderate oxidative and inflammatory effects, whereas 200 nm particles provoked acute cytotoxicity, pronounced endocytosis, and an early inflammatory burst with subdued autophagy. These findings demonstrate that sub-100 nm PS NPs exhibit enhanced skin penetration in barrier-disrupted ex vivo models and induce pronounced oxidative stress, sustained autophagy, and proliferation inhibition in human keratinocytes. While these results suggest potential cellular mechanisms that may contribute to dermal toxicity, they do not directly demonstrate systemic absorption or long-term damage in vivo. Our observations provide a mechanistic basis for future in vivo investigations and highlight the need for caution when extrapolating in vitro findings to human health risks.

## 1. Introduction

Nanoplastics (NPs) have emerged as pervasive environmental pollutants, with polystyrene (PS) being one of the most commonly encountered types due to its widespread use in packaging and consumer products [1,2]. These tiny particles originate from diverse sources, including personal care products, industrial processes, and the degradation of larger plastic debris [3,4]. Due to their stability and persistence in the environment, these particles raise significant concerns regarding their potential impact on human health [5].

Human exposure to plastic particles occurs inevitably via ingestion, inhalation, and dermal contact. Among these routes, dermal contact—long underestimated—requires urgent mechanistic clarification [1]. Human skin is constantly exposed to environmental plastic particles, as it serves as the primary barrier against pathogens, chemicals, and particles [6]. Owing to their small size, NPs possess a large surface area and high surface energy, which enables their potential for deep biological and vital organ penetration [7]. For instance, systematic comparisons using porcine skin models have shown that 20 nm polystyrene nanoplastics (PS NPs) accumulated preferentially in hair follicles and exhibited significantly higher permeation through the stratum corneum compared to their 200 nm counterparts, suggesting an enhanced risk of systemic absorption [7,8]. Beyond physical penetration, emerging evidence highlights that NPs can induce various biological effects, including oxidative stress, inflammation, and autophagy [9,10]. Mechanistically, PS NPs has been found to induce oxidative stress and inflammatory responses, leading to cell death and epithelial barrier dysfunction, which may contribute to tissue damage and pulmonary disorders upon chronic exposure [11]. Similarly, research has reported that PS MPs can inhibit cell viability, elevate ROS production, and trigger apoptosis through a mitochondria-dependent pathway [12].

The toxicity of NPs exhibits pronounced size-dependent characteristics, primarily governed by the surface effect theory. As particle size decreases, the exponential increase in specific surface area amplifies interactions with biological membranes, thereby elevating bioactivity and toxicity risks [4]. This size-dependent toxicity has also been corroborated in pulmonary models. A recent study combining in vivo and in vitro approaches demonstrated that polystyrene microplastics induced oxidative stress, mitochondrial dysfunction, and apoptosis in lung cells in a strict size-dependent manner (1 µm > 5 µm > 10 µm), with smaller particles exhibiting greater oxidative stress and pathological injury, while larger particles triggered distinct cellular responses via the ECM–MMP pathway [13]. For dermal exposure, the sub-100 nm size range is of particular concern. Particles below 100 nm not only achieve deeper skin penetration and higher cellular uptake but also possess the physical capability to enter the nucleus and interact with genetic material. As highlighted in a recent review, intracellular nanoplastics can induce genotoxic and neurotoxic effects, raising the possibility of long-term, non-reversible health consequences [14]. This unique hazard of sub-100 nm NPs—beyond mere oxidative or inflammatory stress—makes understanding their toxicological behavior a critical priority for environmental and dermal risk assessment. It is important to note, however, that the relationship is not universally linear across all toxicological endpoints. A study on HepG2 cells revealed a more complex situation: although smaller PS NPs (50–200 nm) exhibited higher overall toxicity, larger particles (500–5000 nm) specifically triggered a greater degree of apoptosis [15]. This indicates that NP size can differentially influence various cytotoxic pathways. When extrapolating these mechanistic insights to human risk assessment, significant caution is required due to interspecies differences, highlighting the necessity for human-derived research models [16]. Current research on NPs has predominantly focused on aquatic organisms, leaving significant gaps in our understanding of their effects on human skin cells. A recent study utilized ex vivo human skin and 3D co-culture models to demonstrate that barrier disruption enhances NP penetration and that NP accumulation can trigger an IL-17-mediated inflammatory response in keratinocytes [17]. Similarly, the internalization of 100 nm PS NPs by HaCaT cells without significant acute cytotoxicity has been reported [18], while further work has shown that short- and long-term exposure to PS NPs (primarily 100 nm and larger) promotes oxidative stress and divergently affects skin cell signaling [19]. Despite these important contributions, existing studies have predominantly focused on single particle sizes (often 100 nm or larger) or have not systematically dissected how particle size orchestrates the interplay between endocytic uptake, oxidative stress, inflammation, and autophagy over time. Specifically, the differential toxicological behavior of sub-100 nm particles (e.g., 50 nm) versus 100 nm and 200 nm particles within the same keratinocyte model remains poorly characterized. Therefore, this study aimed to systematically investigate the size-dependent toxic effects of 50, 100, and 200 nm PS NPs on HaCaT cells, with a particular focus on comparing their cellular uptake efficiency, oxidative stress induction via the Nrf2 pathway, inflammatory cytokine profiles, and autophagic flux. By integrating these endpoints, we sought to identify the distinct size-dependent toxicity mechanisms that may underlie the dermal health risks of nanoplastics.

## 2. Materials and Methods

### 2.1. Cell Culture and Reagents

HaCaT cells were purchased from the National Infrastructure of Cell Line Resource (Beijing, China) and cultured in DMEM medium containing 10% fetal bovine serum (FBS) and 1% penicillin/streptomycin. The cultures were maintained at 37 °C in a humidified incubator with 5% CO_2_. Phosphate-buffered saline (PBS) and trypsin-EDTA were purchased from Biorigin (Beijing, China). PS NPs with the sizes of 50, 100, and 200 nm were purchased from Polysciences (Warrington, PA, USA). Three fluorescently labeled PS NPs were obtained from Shanghai Huizhi Bio-Tech (Shanghai, China). 3-(4,5-Dimethylthiazol-2-yl)-2,5-diphenyltetrazolium bromide (MTT) was obtained from Sigma-Aldrich (St. Louis, MO, USA). Anti-rabbit NQO1, anti-rabbit Nrf2, anti-mouse Keap1 antibodies, anti-mouse Heme Oxygenase 1, and anti-mouse m-IgGκ horseradish peroxidase-conjugated (HRP) secondary antibody were purchased from Santa Cruz (Dallas, TX, USA). A fluorescently labeled goat anti-rabbit IgG secondary antibody was purchased from Abcam (Cambridge, UK). Anti-rabbit LC3, anti-mouse P62, anti-rabbit ATG5, anti-rabbit Beclin-1, and anti-mouse mTOR secondary antibodies were purchased from Abmart (Shanghai, China). Eea-1 secondary antibody was purchased from Cell Signaling Technology (Danvers, MA, USA). For the skin penetration studies, full-thickness porcine skin samples obtained from Bama miniature pigs were purchased from Taizhou Taihe Biotechnology (Taizhou, China).

### 2.2. Characterization of PS NPs

Suspensions of PS NPs at three distinct sizes were prepared at 50 μg/mL in PBS and 500 μg/mL in complete culture medium containing 90% DMEM, 10% FBS, and 1% penicillin/streptomycin. For morphological characterization, PBS-based suspensions were deposited onto silicon wafers, dried, and analyzed via Zeiss Merlin Compact SEM (Carl Zeiss AG, Oberkochen, Germany) at a scale of 20–200 nm. Culture medium-based suspensions were sonicated for 30 min, incubated for 24 h in an incubator at 37 °C with 5% CO_2_, and subjected to particle size measurements at 0 and 24 h using a Zetasizer Nano ZS90 (Malvern Panalytical, Malvern, UK). All measurements were performed at 25 °C with a detection angle of 90°, preceded by 30 min ultrasonication to eliminate aggregation effects.

### 2.3. In Vitro Skin Permeation Test

Porcine skin samples were divided into two groups: intact skin and barrier-disrupted skin damaged by 15 layers of tape stripping. Each skin piece was rinsed with PBS, oriented with the stratum corneum facing upward, and mounted between the donor and receptor compartments of vertical diffusion cells (effective diffusion area = 7.068 cm^2^). The receptor compartment was filled with 7–8 mL of PBS (pH 7.4) and pre-equilibrated at 32 ± 1 °C in a water bath for 30 min to remove air bubbles. Three fluorescently labeled PS NPs suspensions (5 μg/cm^2^) were added to the donor compartment. The assembled diffusion cells were incubated at 32 ± 1 °C with continuous stirring at 800 rpm. After 1, 4, and 8 h, the skin samples were removed and divided: one portion was wrapped in plastic film and aluminum foil for light protection and stored at −80 °C, while the other was stored at 4 °C. For cryosectioning, frozen skin samples at −80 °C were sectioned using a CryoStar NX50 cryostat microtome (Thermo Fisher Scientific, Waltham, MA, USA) mounted on slides, and observed under a TH4-200 inverted fluorescence microscope (Olympus, Tokyo, Japan) to analyze particle penetration. The samples stored at 4 °C were analyzed via thick-tissue z-stack scanning using a confocal laser scanning microscope (ECLIPSE Ti, Nikon, Tokyo, Japan) to assess morphological changes and particle localization.

### 2.4. Cell Viability Assay

Cytotoxicity was assessed using the MTT assay. HaCaT cells were seeded into 96-well plates at a density of 8 × 10^3^ cells per well and cultured for 24 h. After the medium was removed, the cells were treated with PS NPs of different particle sizes at concentrations ranging from 12.5 to 2000 μg/mL for an additional 24 h. Subsequently, 10 µL of MTT solution (5 mg/mL) was added to each well, followed by incubation at 37 °C for 4 h. After incubation, 100 μL of lysis solution containing 5% isobutanol, 10% SDS, and 12 mM HCl in H_2_O was added to each well to solubilize the formazan crystals. Absorbance was measured at 570 nm using an Infinite M200 Pro multimode microplate reader (Tecan, Männedorf, Switzerland).

### 2.5. Assessment of Cell Proliferation by 5-Ethynyl-2′-Deoxyuridine (EdU) Assay

Cell Proliferation was assessed using an EdU assay Kit (Beyotime, Shanghai, China). HaCaT cells were cultured in 24-well plates at a density of 6 × 10^4^ cells per well and treated with PS NPs of different particle sizes at concentrations of 25, 50, and 500 μg/mL for 24, 48, and 72 h. Next, 500 μL EdU solution was added to HaCaT cells and incubated for another 2 h. Following 15 min of immobilization with 4% paraformaldehyde and 10 min of permeation with PBS containing 0.3% Triton X-100 (Biorigin, Beijing, China), cells were fixed with methanol for 20 min at room temperature. After fixation, the cells were incubated with 250 μL Click reaction cocktail for 30 min at room temperature in the dark. Subsequently, the cells were incubated with 100 μL of streptavidin-HRP working solution per well for 30 min at room temperature. Following a washing step, the cells were stained with 150 μL of 3,3′-diaminobenzidine (DAB) substrate solution and incubated for 15 min at room temperature. Images were captured using a TH4-200 inverted fluorescence microscope (Olympus, Tokyo, Japan) to quantify EdU-positive cells.

### 2.6. Transcriptomics

HaCaT cells were seeded in 10 cm cell culture dishes at a density of 1 × 10^5^ cells per well and cultured with 500 µg/mL of PS NPs of varying sizes (50, 100, or 200 nm) for 72 h. After incubation, the cells were transferred into 1.5 mL non-enzymatic centrifuge tubes and centrifuged at 8000 rcf for 2 min at 4 °C. The supernatant was discarded, and the pellet was resuspended. Subsequently, 1 mL of TransZol Up was added to each tube, thoroughly mixed, and stored at −80 °C. Then, RNA was extracted for analysis. RNA sequencing and subsequent bioinformatic analysis were performed by a commercial service provider (Novogene, Beijing, China). Briefly, the Illumina platform was used for sequencing data filtering and assembly, while edgeR was employed to identify differentially expressed genes (DEGs). Volcano plots of DEGs and functional enrichment analysis were performed using Magic Novogene, with Gene Ontology (GO) database annotations. Each treatment was performed in triplicate with three independent biological replicates.

### 2.7. Determination of Intracellular Reactive Oxygen Species (ROS)

ROS generation was evaluated using the fluorescence probe 6-carboxy-2′,7′-dichlorodihydrofluorescein diacetate (DCFH-DA; Beyotime, Shanghai, China). In brief, HaCaT cells were cultured at 1 × 10^5^ cells/well in a six-well plate. After 24 h, the cells were co-incubated with PS NPs of different particle sizes (50, 100, and 200 nm) at concentrations of 25, 50, and 500 μg/mL in an incubator for 24, 48, and 72 h. The DCFH-DA fluorescent probe was diluted in serum-free medium at 1:1000 to a final concentration 10 μmol/L. Following treatment, cells were washed twice with PBS and incubated with 1 mL of the diluted probe per well in the dark at 37 °C for 20 min. After washing to remove excess probe, intracellular ROS levels were quantified by measuring the fluorescence intensity via flow cytometry (Accuri C6, BD Biosciences, San Jose, CA, USA).

### 2.8. Determination of Intracellular Antioxidant Enzyme Activities and Malondialdehyde Level

HaCaT cells were cultured at 1 × 10^5^ cells/well in a six-well plate. After 24 h, the cells were co-incubated with PS NPs of different particle sizes (50, 100, and 200 nm) at concentrations of 25, 50, and 500 μg/mL in an incubator for 24, 48, and 72 h. After co-incubating with PS NPs, the cells were harvested, lysed with Western and IP cell lysis buffer (Beyotime, Shanghai, China) and then centrifuged at 4 °C and 14,000 rcf for 5 min. The resulting supernatant was collected as the test sample for subsequent analysis. The activity of the antioxidant enzymes catalase (CAT), glutathione peroxidase (GSH-Px), and superoxide dismutase (SOD), along with the level of the lipid peroxidation product malondialdehyde (MDA), was evaluated using the indicated kits (Beyotime, Shanghai, China) according to the manufacturer’s instructions.

### 2.9. Quantification of Inflammatory Cytokines Secretion

HaCaT cells were cultured in six-well plates at a density of 1 × 10^5^ cells per well. After 24 h, the cells were treated with PS NPs of varying sizes (50, 100, and 200 nm) at concentrations of 25, 50, and 500 μg/mL for 24, 48, and 72 h. Following incubation, the cell culture supernatants were collected. The concentrations of secreted interleukin-8 (IL-8), interleukin-1β (IL-1β), and tumour necrosis factor-α (TNF-α) were quantified using commercial human enzyme-linked immunosorbent assay (ELISA) kits (Thermo Fisher Scientific, Waltham, MA, USA) according to the protocols provided by the manufacturer.

### 2.10. Determination of NPs Internalization

Cells at a density of 2 × 10^4^ cells/well were grown in 24-well plates containing cell climbing slides. All groups were treated with fluorescently labeled PS-NPs (50, 100, or 200 nm) at a final concentration of 500 µg/mL for 48 h. After discarding the supernatant, the cells were sequentially washed three times with 0.5 mL PBS under dark conditions. Subsequently, cells were fixed with 4% paraformaldehyde for 15 min and stained with 1 μg/mL 4′,6-diamidino-2-phenylindole (DAPI) for 10 min to label the nuclei. Coverslips were then mounted onto slides and the images were captured using a fluorescence microscope (Olympus, Tokyo, Japan).

To validate internalization dynamics at the ultrastructural level, cells were seeded in 6-well plates at a density of 1 × 10^5^ cells per well. All groups were treated with both unlabeled and fluorescently labeled PS NPs at a concentration of 500 μg/mL and incubated for 72 h. After incubation, the cells were harvested into 1.5 mL EP tubes and subjected to centrifugation at 2000 rpm for 5 min at room temperature. After centrifugation, the supernatant was discarded and 1.4 mL ice-cold 2.5% glutaraldehyde (4 °C) was slowly added along the tube wall, followed by overnight fixation at 4 °C. Following overnight fixation at 4 °C, samples were mounted on grids, dried, and examined using a JEM-F200 TEM (JEOL, Tokyo, Japan) under standard imaging conditions.

### 2.11. Real-Time PCR Measurement

Total RNA was isolated using TRIzol reagent (Invitrogen, Carlsbad, CA, USA). Complementary DNA (cDNA) was synthesized from the RNA using the ReverTra Ace qPCR RT kit (TOYOBO, Shanghai, China) following the manufacturer’s instructions. Quantitative real-time PCR (qPCR) was carried out on the LightCycler^®^ 480 II system sourced from Roche (Basel, Switzerland), where SYBR Green Real-time PCR Master Mix (TOYOBO, Shanghai, China) was employed according to standardized protocols. All primer sequences are listed in Table S1 and were synthesized by Sangon Biotech (Shanghai, China). The Ct values served as the basis for real-time quantification, and the 2^−∆∆Ct^ method was used to calculate gene expression levels that were adjusted to β-actin.

### 2.12. Western Blot Analysis

Cells were lysed with Western and IP cell lysis buffer (Beyotime, Shanghai, China), and cell debris was removed via centrifugation. The protein concentration in the supernatant was quantified with a bicinchoninic acid (BCA) assay kit (Beyotime, Shanghai, China). Equal amounts of protein were loaded onto sodium dodecyl sulfate-polyacrylamide gel electrophoresis (SDS-PAGE; Beyotime, Shanghai, China) after quantification, and subsequently transferred to polyvinylidene difluoride (PVDF) membranes (Beyotime, Shanghai, China). Membranes were blocked with a QuickBlock™ Western Occluder (Beyotime, Shanghai, China) and incubated with the primary antibody overnight at 4 °C. The membranes were then treated with a horseradish peroxidase-conjugated (HRP) secondary antibody (Santa Cruz Biotechnology, Dallas, TX, USA) for 2 h at a diluted concentration of 1:5000 and were visualized using a Tanon 5200 Multi chemiluminescence imaging system (Tanon, Shanghai, China). The proteins were quantified using the ImageJ software (version 1.54g, National Institutes of Health, Bethesda, MD, USA) and normalized to β-actin. All experiments were performed in at least three independent replicates to ensure reproducibility.

### 2.13. Immunofluorescence Analysis

The cells were washed with PBS and then fixed with 4% paraformaldehyde for 15 min. Subsequently, cells were washed three times with PBS (5 min each wash) and permeabilized with 0.3% Triton X-100 in PBS for 10 min. After three additional washes, the cells were blocked with 1% bovine serum albumin (BSA) in PBS for 2 h at room temperature and incubated with primary antibodies overnight at 4 °C. Following three washes with PBS, cells were incubated with fluorophore-conjugated secondary antibodies for 2 h at room temperature. DAPI was used to label the nuclei, and images were obtained with a TH4-200 inverted fluorescence microscope (Olympus, Tokyo, Japan).

### 2.14. Statistical Analysis

Statistical analyses were performed using GraphPad Prism software version 8.0.2 (Dotmatics, Boston, MA, USA). Data are presented as the means  ±  standard deviation (SD) from three independent experiments. To compare the hydrodynamic diameter of PS NPs in cell culture medium at 0 h and 24 h, a paired *t*-test was used. Differences between two experimental factors were assessed via two-way analysis of variance (ANOVA). Comparisons among multiple treatment groups and a single control group were performed using one-way ANOVA followed by Dunnett’s post hoc test. A *p*-value of less than 0.05 was considered statistically significant. For the cell viability assay, six independent replicates (*n* = 6) were used to ensure sufficient statistical power for detecting small differences; for all other assays, three independent replicates (*n* = 3) were performed, each with technical triplicates.

## 3. Results

### 3.1. Characteristics of PS NPs

As shown in Figure S1, the PS NPs of three distinct sizes exhibited uniform dispersion and a regular spherical morphology, but were also prone to agglomeration. The 50 nm and 100 nm PS NPs demonstrated relatively uniform size distributions, whereas the 200 nm PS NP sample contained a significant number of smaller particles. As summarized in Table S2, the hydrodynamic diameter of all three PS NP types increased slightly after 24 h in cell culture, although this change was only statistically significant for the 200 nm PS NPs. The 50 nm and 100 nm PS NPs showed no significant size alteration, indicating greater stability under the conditions tested. Regarding the polydispersity index (PDI) and zeta potential, no statistically significant differences were observed after 24 h; nevertheless, both parameters exhibited slight trends—PDI remained below 0.3, and the absolute zeta potential values stayed below 30 mV, with a minor decrease compared to 0 h. These results suggest that the three PS NP types maintained a relatively stable state in the culture medium.

### 3.2. PS NPs Exhibited Size- and Barrier-Dependent Penetration Profiles

**Figure 1:**
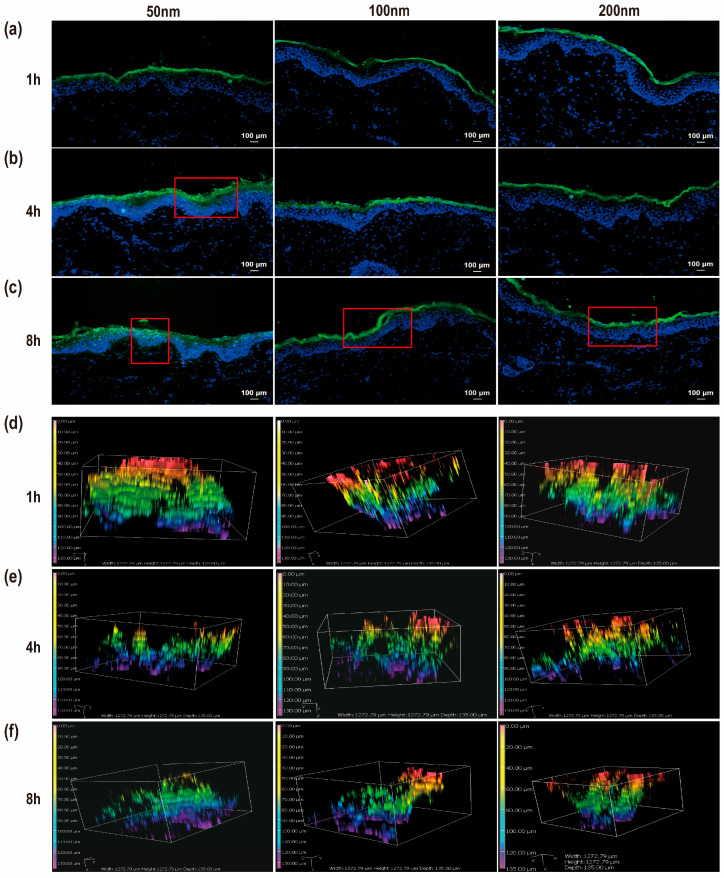
Penetration of polystyrene nanoparticles (PS NPs) of different sizes (50, 100, and 200 nm) into barrier-disrupted porcine skin over different exposure durations (1, 4, and 8 h), assessed via fluorescence and confocal microscopy. (**a**–**c**) Fluorescence microscopy images showing PS NP penetration patterns (green) with DAPI-stained cell nuclei (blue) at 1 h (**a**), 4 h (**b**), and 8 h (**c**). Images are representative of three independent experiments (*n* = 3). The red rectangles indicate the penetration of PS NPs through the stratum corneum. Scale bars: 100 μm. (**d**–**f**) Representative confocal Z-stack images illustrating the spatial distribution of PS NPs following dermal exposure at 1 h (**d**), 4 h (**e**), and 8 h (**f**). Superficial layers are shown in red and deeper layers in purple. Images are representative of three independent experiments (*n* = 3). Scale bars: 100 μm.

Fluorescence imaging revealed distinct penetration profiles of PS NPs in normal versus barrier-impaired skin models. In normal skin, PS NPs of all three sizes were confined to the superficial stratum corneum layers, with no enhancement in penetration observed over time (Figure S2a–c). In contrast, within barrier-disrupted skin, fluorescence signals progressively intensified with longer exposure durations. After 8 h, 50 nm PS NPs had penetrated into the deep stratum corneum, while both 100 nm and 200 nm PS NPs showed significantly greater penetration depth compared to normal skin (Figure 1c). These results demonstrate that PS NPs penetrate the stratum corneum more readily when the skin barrier is compromised. Confocal Z-stack reconstructions of normal skin confirmed that PS NP signals were predominantly localized to the upper layers, with a gradual increase in penetration depth over time (Figure S2d–f). Analysis of the fluorescence patterns indicated that PS NPs primarily entered via hair follicles. Among them, 50 nm PS NPs exhibited the strongest penetration capability, although a portion remained on the skin surface even after 8 h of exposure (Figure S2f). Conversely, in barrier-disrupted skin, the majority of PS NPs had penetrated into the skin after exposure times exceeding 4 h (Figure 1f). Notably, nearly all 50 nm PS NPs entered the skin through hair follicles, while most 100 nm and 200 nm NPs exhibited the strongest penetration capability, with only a small fraction remaining on the skin surface.

### 3.3. PS NPs Exerted Size-Dependent Inhibitory Effects on HaCaT Cell Proliferation

**Figure 2:**
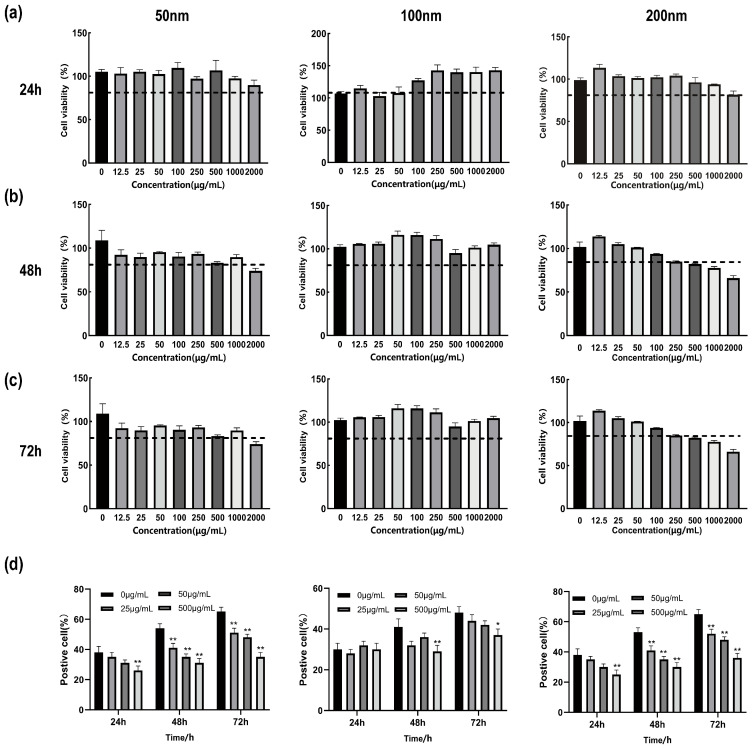
Differential effects of polystyrene nanoparticles (PS NPs) of different sizes on HaCaT cell viability and proliferation. Cell viability assessed via MTT assay after exposure to 50 nm, 100 nm, or 200 nm PS NPs for 24 (**a**), 48 (**b**), and 72 h (**c**). The dashed line indicates the 80% viability threshold. Statistical significance was determined via one-way ANOVA followed by Dunnett’s post hoc test. Data are presented as mean ± SD from six independent experiments (*n* = 6). (**d**) Effects of PS NPs on HaCaT cell proliferation. Quantitative analysis of proliferating HaCaT cells after treatment with 0, 25, 50, or 500 μg/mL of 50 nm, 100 nm, and 200 nm PS NPs for 24, 48, and 72 h. Data are presented as mean ± SD from three independent experiments (*n* = 3). Statistical significance was determined via two-way ANOVA followed by Dunnett’s post hoc test. * *p* < 0.05, ** *p* < 0.01 compared with the control (0 μg/mL) group at each time point.

As shown in Figure 2a, both 50 nm and 100 nm PS NPs exhibited negligible cytotoxicity across all tested concentrations (12.5–2000 μg/mL) at 24 h, with cell viability maintained above 90%. Notably, 100 nm particles even stimulated mild proliferation at concentrations exceeding 100 μg/mL after 24 h. For 50 nm particles, a transient reduction in viability was observed at 48 h for concentrations above 500 μg/mL, though complete recovery occurred by 72 h (Figure 2b,c). In contrast, 200 nm PS MPs induced dose-dependent cytotoxicity, particularly after prolonged exposure (48–72 h), where viability fell below 80% at 2000 μg/mL (Figure 2c). Intriguingly, a transient decline in viability at 48 h, followed by recovery to above 90% at 72 h, was observed for both 50 nm and 200 nm groups. This pattern suggests potential activation of cytoprotective pathways during prolonged exposure. Based on these differential responses, subsequent experiments employed representative concentrations of 25, 50, and 500 μg/mL.

As shown in Figure 2d, exposure to both 50 nm and 200 nm PS NPs significantly inhibited the proliferation of HaCaT cells at all three tested concentrations after both 48 and 72 h, with a marked reduction in proliferating cells. In contrast, 100 nm PS NPs significantly inhibited proliferation only at the high concentration of 500 μg/mL after 48 and 72 h. Following 24 h of exposure, 50 nm and 200 nm PS NPs at 500 μg/mL significantly suppressed proliferation, whereas 100 nm particles showed no inhibitory effect at this time point. The inhibitory effects on proliferation were found to be concentration- and time-dependent, becoming more pronounced with higher concentrations and longer exposure durations. Overall, 50 nm and 200 nm particles exhibited significantly stronger inhibition of cell proliferation than 100 nm particles.

### 3.4. PS NPs Altered Gene Expression in Inflammation, Autophagy and Glutathione Pathways in HaCaT Cells

**Figure 3:**
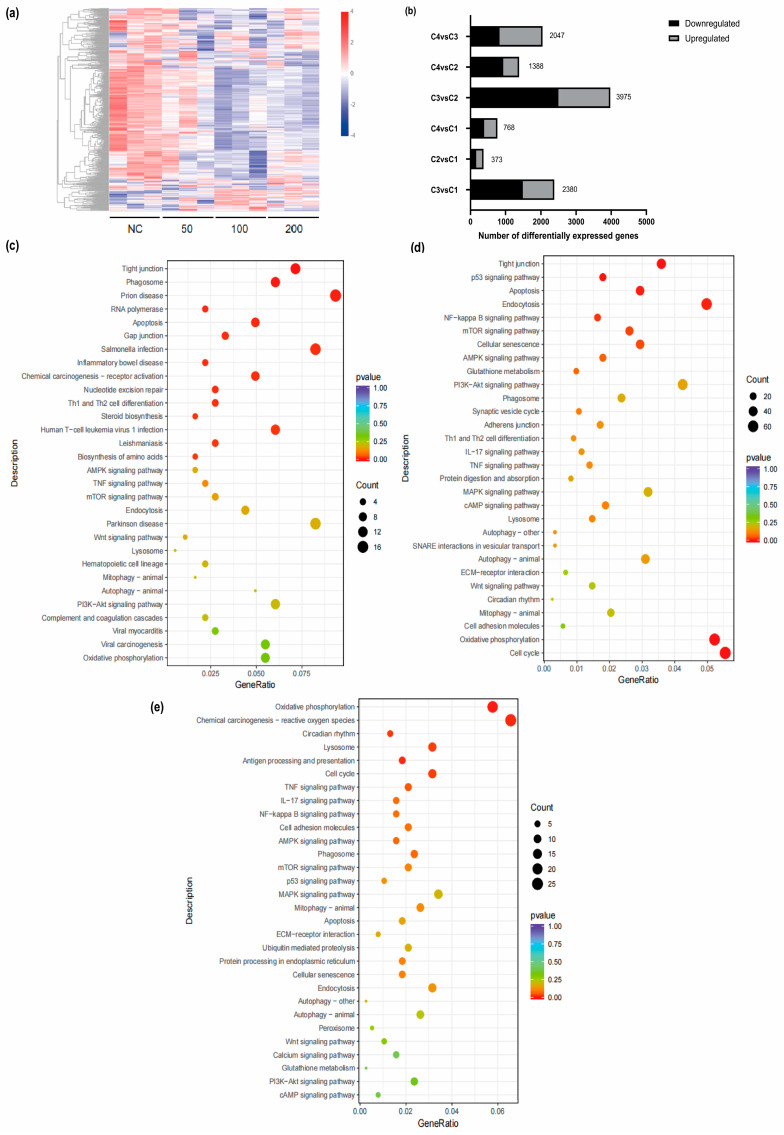
Transcriptomic analysis of differentially expressed genes in HaCaT cells exposed to PS NPs. (**a**) Hierarchical clustering heatmap of DEGs across experimental groups (C1: untreated control; C2: 50 nm; C3: 100 nm; C4: 200 nm PS NPs). Each row represents a gene, and columns represent biological replicates per group. Expression levels are normalized and color-coded (red: up-regulation; blue: down-regulation). (**b**) Bar chart showing the number of significantly up-regulated (red) and down-regulated (blue) DEGs for each comparison relative to the C1 control (adjusted *p*-value ≤ 0.05, |log2fold-change| ≥ 1). (**c**–**e**) Scatter plots of the top significantly enriched KEGG pathways (adjusted *p* ≤ 0.05) for the comparisons (**c**) C2 vs. C1, (**d**) C3 vs. C1, (**e**) C4 vs. C1. KEGG, Kyoto Encyclopedia of Genes and Genomes.

Transcriptomic analysis revealed that treatment with PS NPs of three sizes significantly altered the gene expression patterns in HaCaT cells compared to the control group (C1), with distinct differences in the degree and pattern of expression perturbation depending on particle size (Figure 3a). Analysis of differentially expressed genes (DEGs) showed that 100 nm PS NPs (C3) induced the greatest transcriptional change, followed by 200 nm (C4) and 50 nm (C2) particles (Figure 3b). Compared to the C1 control, 373 (165 down-regulated and 208 up-regulated), 2380 (1491 down-regulated and 889 up-regulated), and 768 (391 down-regulated and 377 up-regulated) DEGs were identified in the C2, C3, and C4 groups, respectively. Substantial differences were also observed in direct comparisons between treated groups: 3975 DEGs between the C3 vs. C2, 2047 DEGs between the C4 vs. C3, and 1388 DEGs between the C4 vs. C2. These results suggested that exposure to PS NPs significantly affects the gene expression profile of HaCaT cells, and the extent of this impact strongly correlates with particle size. Kyoto Encyclopedia of Genes and Genomes (KEGG) pathway enrichment analysis of the DEGs is presented in scatter plots (Figure 3c–e). The significantly enriched pathways were primarily associated with inflammation (TNF, p53, NF-κB), autophagy, endocytosis, glutathione metabolism, apoptosis, and cell cycle. Based on these transcriptomic findings, the oxidative stress, cellular endocytosis, and autophagy pathways were selected for further validation. For instance, upregulation of genes involved in glutathione metabolism and Nrf2 signaling (e.g., HO-1, NQO1) aligned with subsequent oxidative stress measurements, while increased expression of autophagy-related genes (ATG5, p62, LC3B) corresponded to the autophagic phenotype observed via TEM and Western blot.

### 3.5. PS NPs Induced Size-Dependent Oxidative Stress in HaCaT Cells

**Figure 4:**
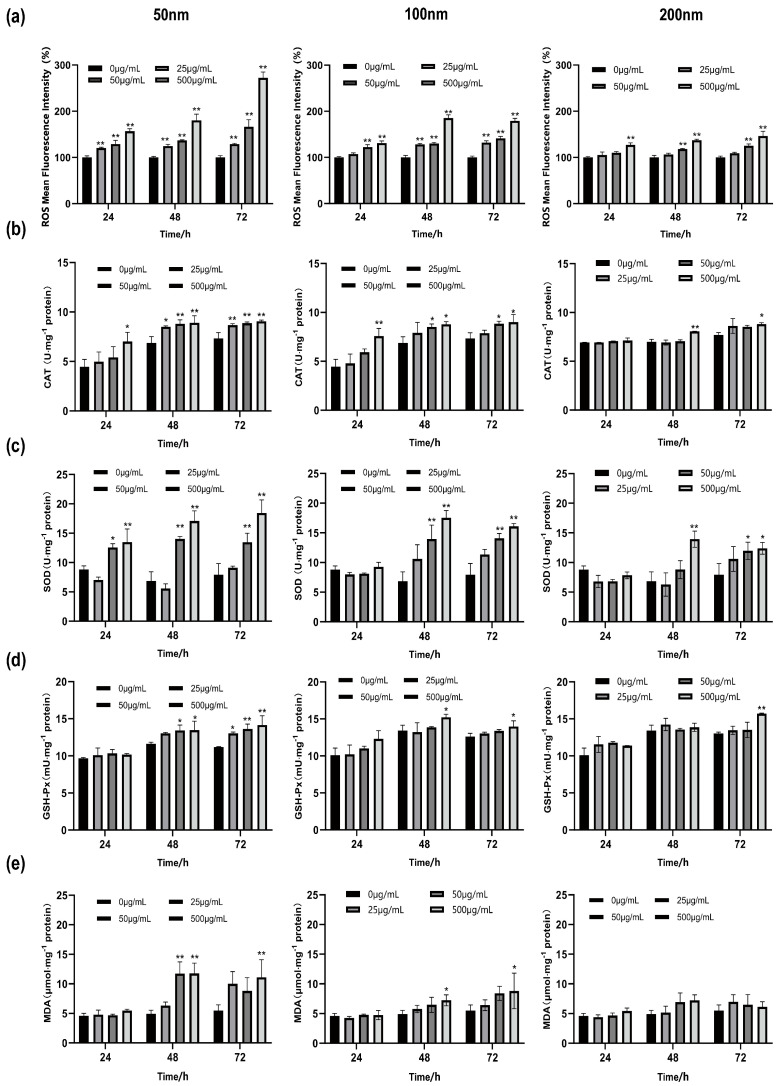
Effects of PS NPs on oxidative stress and antioxidant responses in HaCaT cells. (**a**) Quantitative analysis of intracellular reactive oxygen species (ROS) production. Bar graphs show ROS levels (mean fluorescence intensity, MFI) expressed as a percentage of untreated controls in cells exposed to 50 nm, 100 nm, and 200 nm polystyrene nanoparticles (PS NPs) at concentrations of 0, 25, 50, and 500 μg/mL for 24, 48, and 72 h. (**b**–**e**) Effects of PS NPs on antioxidant enzyme activities and lipid peroxidation, grouped by particle size. Under the same concentration and time conditions, catalase (CAT) activity (**b**), superoxide dismutase (SOD) activity (**c**), glutathione peroxidase (GSH-Px) activity (**d**), and malondialdehyde (MDA) levels (**e**) were measured. Data for 50 nm, 100 nm, and 200 nm PS NPs are shown. Data are presented as mean ± SD from three independent experiments. Statistical significance was determined via two-way ANOVA followed by Dunnett’s post hoc test. * *p* < 0.05, ** *p* < 0.01 versus the control group (0 μg/mL) at the corresponding time point and for the same particle size.

Compared to the control group, 50 nm and 100 nm PS NPs induced a significant, dose-dependent increase in ROS fluorescence intensity across all tested concentrations over 72 h, with the 50 nm particles exhibiting the most pronounced effect (Figure 4a). In contrast, 200 nm PS NPs triggered a significant ROS increase only at the higher concentrations of 50 and 500 μg/mL (Figure 4a). For all particle sizes, ROS levels exhibited a time-dependent escalation with prolonged exposure. Prolonged exposure to PS NPs induced time-dependent variations in the activities of key antioxidant enzymes. Following 48–72 h treatment, the activities of SOD and CAT showed concentration-dependent elevation (Figure 4b,c). GSH-Px activity was significantly increased by 50 nm PS NPs and by high concentrations (500 μg/mL) of 100 nm and 200 nm PS NPs (Figure 4d). Furthermore, MDA levels exhibited a significant increasing trend in cells treated with 50 nm and 100 nm PS NPs, while the effect of 200 nm PS NPs was less pronounced (Figure 4e). Collectively, 50 nm PS NPs induced the most significant oxidative stress effects, consistent with the ROS results. This indicates that smaller particles possess a greater potential to disrupt cellular redox balance.

### 3.6. PS NPs Triggered Nrf2 Nuclear Translocation and Upregulated Downstream Antioxidant Genes in HaCaT Cells

**Figure 5:**
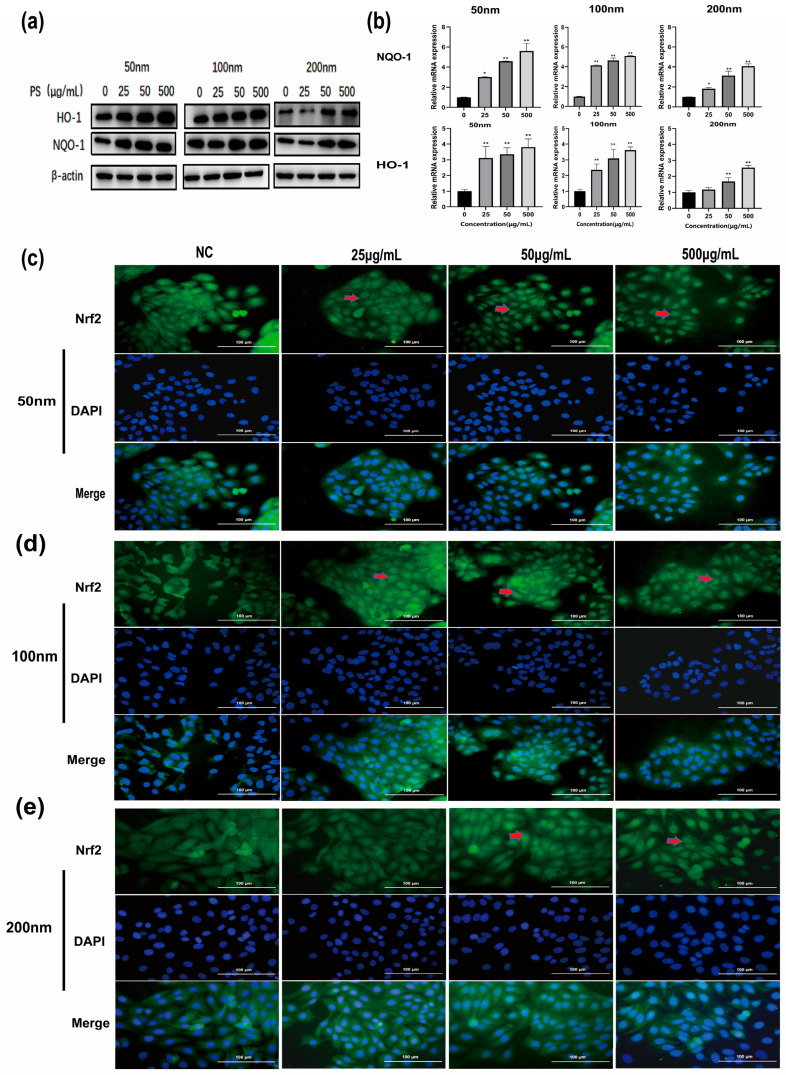
Activation of the Nrf2-mediated antioxidant pathway in HaCaT cells by PS NPs. (**a**) Immunoblot analysis of HO-1 and NQO-1 following 24 h exposure to 50, 100, or 200 nm PS NPs at indicated concentrations (0, 25, 50, 500 µg/mL). Data were normalized to β-actin. The samples derive from the same experiment or parallel experiments and the gels/blots were processed in parallel. (**b**) Quantitative real-time PCR analysis of HO-1 and NQO-1 mRNA expression under the same treatment conditions. Data are normalized to β-actin and presented as mean ± SD from three independent experiments. Statistical significance was determined via one-way ANOVA followed by Dunnett’s post hoc test. * *p* < 0.05, ** *p* < 0.01 compared with the control (0 μg/mL) group for the same particle size. (**c**–**e**) Immunofluorescence analysis of Nrf2 nuclear translocation in HaCaT cells treated with PS NPs of different sizes: 50 nm (**c**), 100 nm (**d**), and 200 nm (**e**) at the indicated concentrations for 24 h. From top to bottom: Nrf2 signal (green), DAPI-stained nuclei (blue), and merged images. Images are representative of three independent experiments (*n* = 3). Red arrows indicate nuclear accumulation of Nrf2. Scale bars: 100 μm.

Exposure of HaCaT cells to three distinct sizes of PS NPs for 24 h significantly upregulated mRNA and protein expression levels of the downstream antioxidant genes HO-1 and NQO-1 in a dose-dependent manner (Figure 5b and Figure S3). Notably, smaller particles (50 nm) exhibited a more pronounced upregulation. Immunofluorescence analysis further confirmed the progressive nuclear translocation of Nrf2 protein from the cytoplasm following PS NP treatment (Figure 5c–e). These results demonstrate that PS NPs activate the Nrf2 signaling pathway, inducing oxidative stress in HaCaT cells. Notably, at non-cytotoxic concentrations, the smaller (50 nm) particles triggered a more pronounced oxidative stress response than their larger counterparts.

### 3.7. PS NPs Stimulated Pro-Inflammatory Cytokine Secretion in HaCaT Cells

As shown in Figure S4, PS NPs of three sizes significantly induced the secretion of IL-8, TNF-α, and IL-1β after 48 h and 72 h of exposure at concentrations exceeding 50 μg/mL. Notably, the most pronounced effect was observed for IL-1β, where a high concentration of PS NPs induced an approximately two-fold increase in secretion compared to untreated controls following 72 h of exposure. At 24 h, while 50 nm and 100 nm PS NPs showed no significant effect on cytokine secretion across all tested concentrations, 200 nm PS NPs at high concentrations significantly upregulated all three cytokines. Collectively, these findings demonstrate that particle size is a key determinant of the inflammatory response, with larger particles causing more pronounced cytokine secretion upon prolonged exposure, potentially contributing to skin inflammation.

### 3.8. PS NPs Entered HaCaT Cells via Endocytosis

**Figure 6:**
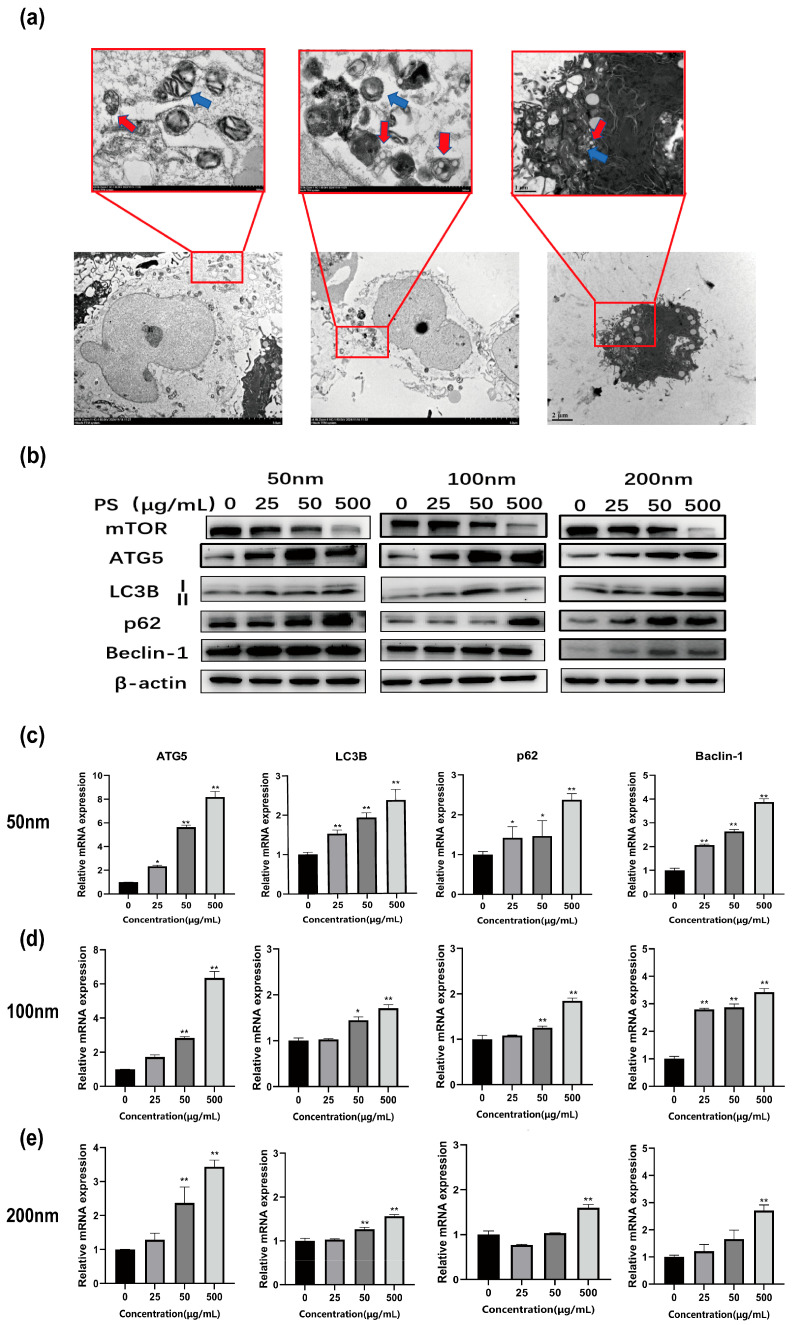
PS NPs induce autophagy in HaCaT cells. (**a**) Representative TEM images of cells after 24 h exposure to 50, 100, or 200 nm PS NPs. Red arrows indicate PS NPs sequestered within autophagosomes. Images are representative of three independent experiments (*n* = 3). Blue arrows indicate autophagosomes. (**b**) Immunoblot analysis of autophagy-related markers (mTOR, LC3B, p62, ATG5, Beclin-1) in HaCaT cells treated with indicated concentrations (0, 25, 50, 500 µg/mL) of PS NPs for 24 h. Data were normalized to β-actin. The samples derive from the same experiment or parallel experiments and the gels/blots were processed in parallel. (**c**–**e**) mRNA expression levels of ATG5, LC3B, p62, and Beclin-1 under the same treatment conditions with different PS NP sizes of 50 (**c**), 100 (**d**), and 200 nm (**e**). Protein levels were quantified and normalized to β-actin. Bar graphs are representative of three independent experiments. Data are presented as mean ± SD (n = 3). Statistical significance was determined via one-way ANOVA followed by Dunnett’s post hoc test. * *p* < 0.05, ** *p* < 0.01 compared to the respective 0 μg/mL control for the same NP size.

Fluorescence microscopy revealed that following 72 h of exposure, fluorescent PS NPs of all three sizes (50, 100, and 200 nm) were internalized and accumulated within the cytoplasm of HaCaT cells (Figure S5a). This visual evidence was corroborated by transmission electron microscopy (TEM), which clearly showed the presence of PS NPs in the cytoplasmic compartment (Figure 6a). Notably, a subset of the internalized NPs appeared to be encapsulated within autophagosome-like structures. To specifically investigate endocytic uptake, the endocytosis inhibitor 2-deoxy-D-glucose (2-DG) was employed. Results showed a significant increase in both mRNA and protein expression levels of the early endosome marker EEA-1 in HaCaT cells exposed to PS NPs alone, which exhibited a remarkable rise at 200 nm (Figure S5b,c). However, co-treatment with 2-DG significantly inhibited this PS NP-induced upregulation of EEA-1. These findings indicate that endocytosis is a primary entry route for PS NPs into HaCaT cells, with the larger 200 nm NPs showing more pronounced endocytosis.

### 3.9. PS NPs Induced Autophagy in a Size-Dependent Manner

TEM confirmed the intracellular presence of PS NPs and revealed the formation of autophagosomes, providing direct morphological evidence of autophagy activation (Figure 6a). To further evaluate the status of autophagy, we analyzed the key molecular markers: mTOR, Beclin-1, ATG5, LC3B, and p62. As shown in Figure 6b–e and Figure S6, exposure to PS NPs at concentrations above 50 μg/mL significantly increased the mRNA and protein levels of Beclin-1, ATG5, LC3B, and p62 in a dose-dependent manner, while concurrently suppressing the protein expression level of mTOR. These results demonstrate that the autophagy pathway was activated. In summary, PS NPs are internalized primarily via endocytosis and subsequently induce autophagy, with a more pronounced response observed for smaller particle sizes.

## 4. Discussion

NPs have emerged as ubiquitous environmental contaminants, with PS being one of the most prevalent types in consumer products. As the body’s primary physical barrier, the skin is consistently exposed to such contaminants, underscoring the need to elucidate their potential toxicological impacts on skin integrity and cellular homeostasis [20]. This study investigated the size- and time-dependent dermatotoxicity of PS NPs by integrating ex vivo and in vitro models. Our findings reveal a complex, non-linear size-dependent toxicity pattern: 50 nm particles triggered strong oxidative stress and autophagy; 100 nm particles induced moderate oxidative stress and inflammation only after prolonged high-concentration exposure; whereas 200 nm particles elicited acute cytotoxicity and early inflammatory responses. Collectively, these results underscore particle size as a key determinant of biological pathway activation, which is essential for accurate health risk assessment.

The penetration patterns of PS NPs varied with particle size and skin integrity. In barrier-disrupted skin, 50 nm NPs exhibited the deepest penetration into the stratum corneum and hair follicles, followed by 100 nm and 200 nm particles (Figure 1). This observation aligns with prior reports of time-dependent increases in the cutaneous distribution of similarly sized PS NPs, emphasizing the permeation advantage of smaller particles [8]. Further supporting the role of follicular pathways, studies have shown that PS NPs (20 nm, 200 nm) accumulate primarily in the stratum corneum and upper hair follicle regions, with detectable fluorescence signals even beyond 20 μm depth, supporting the role of follicles as a significant pathway [21]. Our results suggest that smaller particles (50 nm) exploit both compromised barrier function and follicular routes for enhanced permeation, whereas larger NPs (200 nm) showed limited penetration even after prolonged exposure. Anatomically, hair follicles are densely surrounded by a fine network of dermal lymphatic capillaries, which are the primary conduits for peripheral tissue drainage and early entry into the lymphatic system. Compared with trans-epidermal permeation that must cross the tight epidermal barrier, the follicular route allows PS NPs to bypass the stratum corneum and directly access the perifollicular dermis, where lymphatic vessels are abundantly distributed. Therefore, preferential follicular penetration, especially for 50 nm sub-100 nm NPs, substantially elevates the likelihood of nanoplastics entering dermal lymphatic vessels and subsequently draining into regional lymph nodes [21]. This underscores an increased risk of systemic absorption for sub-100 nm NPs when the epidermal barrier is impaired. Collectively, the penetration data indicate that internalized PS NPs are likely to reside within keratinocytes, with the potential to perturb normal cellular function. Furthermore, given the dense lymphatic network surrounding hair follicles, preferential follicular penetration—especially of 50 nm NPs—may facilitate subsequent entry into dermal lymphatic vessels and regional lymph nodes, a possibility that warrants investigation in future studies.

The cytotoxicity of PS NPs did not exhibit a simple linear correlation with particle size, showing distinct size- and time-dependent patterns consistent with previous nanotoxicology reports [18,22]. The concentrations used in this study (12.5–2000 μg/mL) were selected based on dermatological exposure relevance and standard nanotoxicology practice [23]. While these levels are higher than reported systemic environmental nanoplastic concentrations, it is critical to distinguish between systemic circulation levels and local tissue concentrations at skin exposure sites. Nanoplastics readily become trapped in the stratum corneum and hair follicles, leading to significant local accumulation, especially in barrier-disrupted skin [17]. All subsequent proliferation and mechanistic assays were performed at concentrations ≤ 500 μg/mL—a range consistent with established nanoplastic dermatotoxicity investigations [18,19,20].

Despite the limited overall cytotoxicity observed, EdU assays revealed that both 50 nm and 200 nm PS NPs significantly inhibited cell proliferation through distinct mechanisms. The smaller 50 nm particles exhibit higher propensity for intracellular penetration and accumulation, leading to direct cellular damage and suppressed proliferation [24,25]. Conversely, internalization of larger 200 nm particles relies primarily on energy-dependent endocytosis, which consumes substantial cellular resources and indirectly restricts proliferative capacity [26]. In comparison, 100 nm PS NPs showed the least pronounced effect on cell proliferation, with mild proliferative promotion observed at concentrations above 100 μg/mL.

Transcriptomic analysis revealed that exposure to different-sized PS NPs commonly dysregulated several core cellular pathways, including those mediating inflammation (e.g., TNF and NF-κB signaling), autophagy, endocytosis, and glutathione metabolism (Figure 3c–e). This engagement of a conserved stress response network aligns with prior toxicogenomic studies on MPs. For instance, a transcriptomic and metabolomic study in earthworms exposed to polypropylene (PP) and polyethylene (PE) MPs also identified significant disruptions in glutathione metabolism and immune-related pathways [27]. Similarly, in adult zebrafish, PE and PS MPs were shown to alter the transcription of immune-related genes and induce oxidative stress, leading to tissue damage [28]. Interestingly, 100 nm PS NPs induced the most extensive transcriptomic changes (2380 DEGs, Figure 3b) but exhibited the least cytotoxicity and even transiently promoted proliferation (Figure 2d). This apparent paradox can be explained by the balance between protective and damaging signals. KEGG analysis (Figure 3d) showed that 100 nm NPs prominently upregulated adaptive pathways such as glutathione metabolism, autophagy, and endocytosis, which enhance cellular defense and clearance. While 50 nm and 200 nm NPs activated similar pathways, they also induced stronger oxidative stress (50 nm) or a greater endocytic burden (200 nm), which partially overwhelmed the protective capacity and led to transient or more pronounced functional impairment. In contrast, 100 nm NPs may fall into a size window that effectively engages cytoprotective mechanisms without exceeding the cellular buffering threshold. Thus, a high number of DEGs does not necessarily equate to high toxicity; it may instead reflect a robust yet successful homeostatic response. Although this specific phenomenon has not been directly reported for polystyrene nanoplastics, similar observations exist for other nanoparticle types, where medium-sized particles triggered the strongest transcriptomic responses [29] and sub-cytotoxic doses still induced significant gene expression changes [30]. Such size-dependent dissociation further confirms that nanoparticle toxicity cannot be inferred from DEG counts alone and reinforces the necessity of functional phenotypic validation [18]. Given that oxidative stress is a master upstream regulator of both inflammation and autophagy, we next examined whether PS NP-induced redox imbalance serves as an early trigger of these transcriptional programs.

Oxidative stress, mediated by particle size and exposure time, was identified as a central mechanism of PS NP toxicity. This finding aligns with the mechanistic framework proposed by Zhou et al., who highlighted that smaller MNPs exhibit enhanced cellular uptake, lysosomal accumulation, and downstream oxidative stress and inflammatory signaling as key drivers of size-dependent toxicity across multiple organ systems [31]. Intracellular ROS levels in HaCaT cells increased following exposure to all tested PS NPs, peaking at 48 h and 72 h (Figure 4a), a pattern that correlates with their high potential for cellular interaction and internalization [32]. The most pronounced ROS induction was elicited by 50 nm particles, consistent with their superior cellular penetration, and the effects intensified with dose and time [33]. This aligns with findings in marine copepods, where 50 nm PS beads provoked the strongest oxidative stress response compared to larger counterparts [34]. Correspondingly, the activities of the antioxidant enzymes SOD, CAT, and GSH-Px were significantly elevated after PS NP treatment (Figure 4b–d), indicating a compensatory activation of cellular defense mechanisms against ROS. Overall, 50 nm PS NPs induced the most significant oxidative imbalance, confirming that smaller particles possess a greater capacity to disrupt cellular redox homeostasis. This was further substantiated by the activation of the Nrf2-mediated antioxidant pathway. Nuclear translocation of Nrf2 and the subsequent upregulation of its downstream targets HO-1 and NQO-1 were most pronounced in cells treated with 50 nm PS NPs (Figure 5). This observation is supported by prior research demonstrating a clear size-dependency in Nrf2 pathway activation, wherein smaller PS NPs triggered a more robust upregulation of Nrf2, HO-1, and NQO-1 in skin cells compared to larger particles [19]. The concordance between our oxidative stress metrics and transcriptomic signatures reinforces the conclusion that smaller NPs exhibit heightened oxidative potential.

Secretion of the pro-inflammatory cytokines IL-1β, IL-8, and TNF-α was significantly upregulated in HaCaT cells, particularly following 48–72 h of exposure to 200 nm NPs at high concentrations (Figure S4). This finding is consistent with prior in vitro evidence showing that exposure to 1 μm PS particles can rapidly upregulate the expression of IL-1β and TNF-α in mouse monocyte/macrophage J774A.1 cells [35]. This inflammatory outcome is mechanistically supported by our transcriptomic data, which showed significant enrichment of the TNF and NF-κB signaling pathways (Figure 3). These results demonstrate that PS NPs can elicit a pro-inflammatory response in keratinocytes, with larger particles eliciting a more robust response under specific exposure conditions.

PS NPs were internalized by HaCaT cells primarily via endocytosis, as evidenced by the upregulation of the early endosome marker EEA-1 and the significant inhibition of uptake by the endocytosis inhibitor 2-DG (Figure S5). This is consistent with prior reports in RAW264.7 cells, where similar-sized (30 nm) PS NPs were internalized via endocytosis with concomitant EEA-1 upregulation, indicating early endosome formation [36]. Following internalization, NPs accumulated within autophagosomal structures (Figure 6a). This was accompanied by molecular signatures of autophagy induction, including increased expression of Beclin-1, ATG5, and LC3B, modulation of p62, and suppression of mTOR (Figure 6b–e and Figure S6). Intriguingly, a differential size-dependent relationship was observed between endocytic uptake and autophagic response. While 200 nm NPs exhibited strong endocytic activity, they triggered relatively low autophagy levels. In contrast, 50 nm NPs, despite showing lower endocytic efficiency, induced a significantly stronger autophagic response. The differential autophagic response appears contingent upon distinct intracellular trafficking routes dictated by particle size. Larger NPs (200 nm) are mainly internalized via clathrin-mediated endocytosis and other classical pathways due to size constraints [37]. This pathway directly delivers NPs to endosomes and ultimately to lysosomes for degradation—an efficient and direct process that may inhibit or obviate the need to activate the more complex autophagic pathway for secondary clearance [38]. In contrast, smaller NPs (50 nm) may enter the cytosol via non-classical or passive mechanisms, bypassing immediate endolysosomal capture [39]. Their cytosolic presence is perceived as cellular stress, thereby inducing autophagy to sequester and clear the particles via autophagosome–lysosome fusion [40,41]. Thus, autophagy appears to serve as a key compensatory clearance pathway for smaller NPs, resulting in significantly elevated autophagic activity. These autophagy findings are consistent with the transcriptomic data showing enrichment of autophagy-related pathways, particularly for smaller particles.

This study has certain limitations. First, polystyrene nanospheres serve as a model particulate that cannot represent the complex physicochemical properties and weathering states of real environmental nanoplastics to which skin is actually exposed. Second, the HaCaT monolayer lacks the stratified architecture and barrier function of human skin, and no quantitative dermal permeation data were obtained, limiting the relevance to in vivo dermal exposure. Third, the molecular mechanistic investigation remains preliminary, as cell viability was assessed solely by the MTT assay without orthogonal confirmation, and autophagic flux was not evaluated, leaving the pathway analysis incomplete. Future studies should integrate 3D skin constructs, environmentally representative particle mixtures, multiple complementary assays, and in vivo validation to strengthen the mechanistic and human health relevance.

## 5. Conclusions

**Figure 7:**
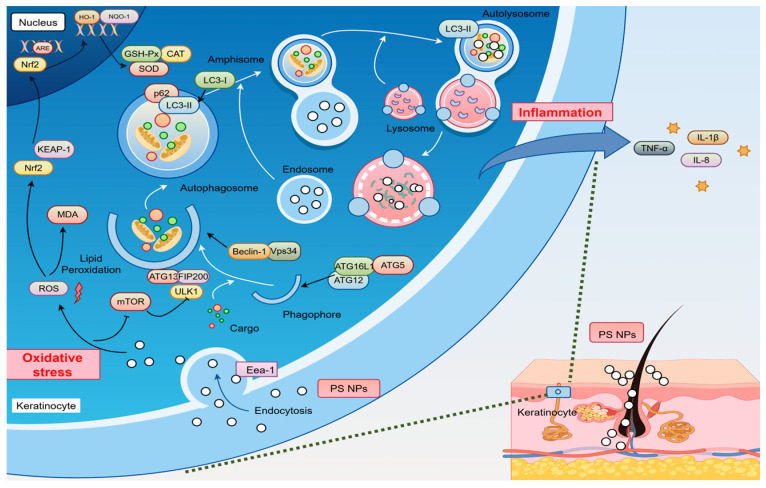
Proposed mechanism of PS NP-induced cellular toxicity in HaCaT cells.

In conclusion, this study reveals non-linear size-dependent dermatotoxicity and distinct mechanistic profiles of polystyrene nanoplastics (50, 100, 200 nm) in HaCaT cells. As illustrated in Figure 7, PS NPs penetrate barrier-disrupted skin mainly via follicular routes, triggering oxidative stress associated with the Nrf2-HO-1/NQO-1 axis, endocytosis, autophagic flux disruption, and pro-inflammatory cytokine secretion. Notably, 50 nm particles show the deepest skin penetration and highest intracellular accumulation, driving the strongest oxidative stress and sustained autophagy, leading to persistent proliferation inhibition and time-dependent inflammation, thus posing the greatest risk of chronic skin damage. In contrast, 100 nm NPs induced the most extensive transcriptional response but exhibited only mild phenotypic effects due to effective cellular homeostatic compensation, even showing transient proliferation under specific conditions. The 200 nm PS NP preparation, which contains a subpopulation of smaller particles, caused acute dose- and time-dependent cytotoxicity, pronounced endocytosis, and early inflammatory bursts at high concentrations, alongside a weaker autophagic response. These findings also indicate that polydisperse PS NP mixtures may exhibit more complex toxicity profiles than ideal monodisperse particles. These in vitro observations provide mechanistic insights into how particle size influences PS NP interactions with skin cells. However, direct extrapolation to human health risks—including systemic absorption or chronic dermal disease—is not supported by the current experimental design. Our findings should therefore be considered hypothesis-generating, identifying key cellular pathways that warrant further investigation in more complex in vivo models and under environmentally relevant exposure scenarios.

## Data Availability

Data will be made available upon request.

## Supplementary Materials

The following supporting information can be downloaded at https://www.mdpi.com/article/10.3390/toxics14060507/s1. Table S1: Quantitative real-time PCR primer sequence; Table S2: The characterization of PS NPs in Cell Culture Medium at 0 and 24 h; Figure S1: Characterization of polystyrene nanoplastics (PS NPs). SEM images of the PS NPs of different sizes (50, 100 and 200nm). Left scale bars: 200 nm, Mag: 40 kx; Right scale bars: 100 nm, Mag: 100 kx; Figure S2: Penetration of polystyrene nanoparticles (PS NPs) of different sizes (50, 100, and 200 nm) into normal porcine skin over different exposure durations (1, 4, and 8 h), assessed by fluorescence and confocal microscopy. (a–c) Fluorescence microscopy images showing PS NP penetration patterns (green) with DAPI-stained cell nuclei (blue) at 1 h (a), 4 h (b), and 8 h (c). Images are representative of three independent experiments (n = 3). Scale bars: 100 μm. (d–f) Representative confocal Z-stack images illustrating the spatial distribution of PS NPs following dermal exposure at 1 h (d), 4 h (e), and 8 h (f). Superficial layers are shown in red and deeper layers in purple. Images are representative of three independent experiments (n = 3). Scale bars: 100 μm; Figure S3: Quantitative analysis of HO-1 and NQO-1 protein expression in HaCaT cells treated with PS NPs of varying sizes. Cells were exposed to 50 nm, 100 nm, or 200 nm PS NPs at indicated concentrations (0, 25, 50, and 500 µg/mL) for 24 h. Protein levels were determined by immunoblotting and normalized to β-actin. (a) Relative expression of HO-1. (b) Relative expression of NQO-1. Bar graphs represent mean ± SD from three independent experiments. Statistical significance was determined by one-way ANOVA followed by Dunnett’s post hoc test. * *p* < 0.05, ** *p* < 0.01 compared with the control group (0 µg/mL) for each particle size; Figure S4: Effect of PS NPs on Pro-inflammatory Cytokine Secretion in HaCaT Cells. Secretion levels of IL-8, IL-1β, and TNF-α were quantified by ELISA in cells treated with 50 nm (a), 100 nm (b), or 200 nm (c) PS NPs at concentrations of 0, 25, 50, or 500 µg/mL for 24, 48, and 72 h. Data are represented as mean ± SD from three independent experiments. Statistical significance was determined by two-way ANOVA followed by Dunnett’s post hoc test. * *p* < 0.05, ** *p* < 0.01 compared with the control (0 µg/mL) at the corresponding time point for the same NP size; Figure S5: Cellular internalization and endocytic uptake of PS NPs in HaCaT cells. (a) Fluorescence microscopy images of HaCaT cells exposed to 50 nm, 100 nm, or 200 nm PS NPs (green) for 72 h. From top to bottom: PS NP signal (green), DAPI-stained nuclei (blue), and merged images. The red arrows indicate PS NPs were internalized and accumulated within the cytoplasm of HaCaT cells. Images are representative of three independent experiments (n = 3). Scale bars: 100 μm. (b,c) Effect of PS NPs and the endocytosis inhibitor 2-deoxy-D-glucose (2-DG) on the early endosomal marker EEA-1. (b) Immunoblot analysis of EEA-1 protein expression. Cells were pretreated with or without the 2-DG prior to exposure to different sizes (50, 100, 200 nm) and concentrations (0, 25, 50, 500 µg/mL) of PS NPs. (c) Corresponding quantitative analysis of EEA-1 protein and mRNA expression levels. Protein levels were normalized to β-actin, and all values were expressed relative to untreated controls (set as 1). The samples derive from the same experiment or parallel experiments and that gels/blots were processed in parallel. Data are represented as mean ± SD from three independent experiments. Statistical significance was determined by one-way ANOVA followed by Dunnett’s post hoc test. ** *p* < 0.01 compared to the PS NP-free group control. ## *p* < 0.01, compared with the corresponding inhibitor-free group within the same treatment condition; Figure S6: Quantitative analysis of autophagy-related markers protein expression in HaCaT cells treated with PS NPs. Cells were exposed to 50 nm (a), 100 nm (b), or 200 nm (c) PS NPs at indicated concentrations (0, 25, 50, and 500 µg/mL) for 24 h. Protein levels of mTOR, LC3B, p62, Beclin-1, and ATG5 were determined by immunoblotting and normalized to β-actin. Bar graphs represent the relative protein expression (mean ± SD) from three independent experiments. Statistical significance was determined by one-way ANOVA followed by Dunnett’s post hoc test. * *p* < 0.05, ** *p* < 0.01 compared with the control group (0 µg/mL) for each particle size.
