# Supplementary material for: The Particle Size Effect: Cytotoxicity and Cellular Uptake of Polystyrene Nanoplastics in Human Keratinocytes
Source: Toxics. 2026 Jun 10;14(6):507. doi: 10.3390/toxics14060507 (PMC13306949; doi:10.3390/toxics14060507)
Supplement: Supplementary file 1 [file toxics-14-00507-s001.zip › toxics-4373181-supplementary.pdf]

Supplementary Material

Table S1. Quantitative real-time PCR primer sequence

Primer	Sequence 5'–3'
β-actin-F	CAGTCGGTTGGAGCGAGCAT
β-actin-R	GGACTTCCTGTAACAACGCATCT
Nrf2-F	AACCAGTGGATCTGCCAACTACTC
Nrf2-R	CTGCGCCAAAAGCTGCAT
Keap1-F	CCTCTGGCCGGGTAATAGG
Keap1-R	CCCCTCCCAGGTATCCAAGA
HO-1-F	CCTCACAGATGGCGTCACTT
HO-1-R	TGGGGGCCAGTATTGCATTT
NQO1-F	GGGCAAGTCCATCCCAACTG
NQO1-R	GCAAGTCAGGGAAGCCTGGA

Table S2. The characterization of PS NPs in Cell Culture Medium at 0 and 24 h

	Hydrodynamic diameter (nm)		PDI		Zeta potential（mV）	
	0 h	24 h	0 h	24 h	0 h	24 h
50 nm	56.4±17.03	61.16±23.3	0.06	0.139	-10.4±2.83	-8.22±4.82
100 nm	99.28±32.49	107.3±27.42	0.081	0.033	-8.96±4.59	-8.81±7.26
200 nm	220.1±75.84	267.1±70.84***	0.127	0.058	-16.1±4.08	-14.7±6.84

Data are presented as mean ± SD (n=3). Statistical significance was determined by a paired t-test; *** $p < 0.001$ compared with the 0 h value for the same particle size.

Figure Caption

Figure S1. Characterization of polystyrene nanoplastics (PS NPs). SEM images of the PS NPs of different sizes (50, 100 and 200nm). Left scale bars: 200 nm, Mag: 40 kx; Right scale bars: 100 nm, Mag: 100 kx.

Figure S2. Penetration of polystyrene nanoparticles (PS NPs) of different sizes (50, 100, and 200 nm) into normal porcine skin over different exposure durations (1, 4, and 8 h), assessed by fluorescence and confocal microscopy. (a-c) Fluorescence microscopy images showing PS NP penetration patterns (green) with DAPI-stained cell nuclei (blue) at 1 h (a), 4 h (b), and 8 h (c) Images are representative of three independent experiments (n = 3). Scale bars: 100 μm. (d-f) Representative confocal Z-stack images illustrating the spatial distribution of PS NPs following dermal exposure at 1 h (d), 4 h (e), and 8 h (f). Superficial layers are shown in red and deeper layers in purple. Images are representative of three independent experiments (n = 3). Scale bars: 100 μm.

Figure S3. Quantitative analysis of HO-1 and NQO-1 protein expression in HaCaT cells treated with PS NPs of varying sizes. Cells were exposed to 50 nm, 100 nm, or 200 nm PS NPs at indicated concentrations (0, 25, 50, and 500 μg/mL) for 24 h. Protein levels were determined by immunoblotting and normalized to β-actin. (a) Relative expression of HO-1. (b) Relative expression of NQO-1. Bar graphs represent mean ± SD from three independent experiments. Statistical significance was determined by one-way ANOVA followed by Dunnett's post hoc test. *$p < 0.05$, **$p < 0.01$ compared with the control group (0 μg/mL) for each particle size.

Figure S4. Effect of PS NPs on Pro-inflammatory Cytokine Secretion in HaCaT Cells. Secretion levels of IL-8, IL-1β, and TNF-α were quantified by ELISA in cells treated with 50 nm

(a), 100 nm (b), or 200 nm (c) PS NPs at concentrations of 0, 25, 50, or 500 μg/mL for 24, 48, and 72 h. Data are represented as mean ± SD from three independent experiments. Statistical significance was determined by two-way ANOVA followed by Dunnett's post hoc test. *$p < 0.05$, **$p < 0.01$ compared with the control (0 μg/mL) at the corresponding time point for the same NP size.

Figure S5. Cellular internalization and endocytic uptake of PS NPs in HaCaT cells. (a) Fluorescence microscopy images of HaCaT cells exposed to 50 nm, 100 nm, or 200 nm PS NPs (green) for 72 h. From top to bottom: PS NP signal (green), DAPI-stained nuclei (blue), and merged images. The red arrows indicate PS NPs were internalized and accumulated within the cytoplasm of HaCaT cells. Images are representative of three independent experiments (n = 3). Scale bars: 100 μm. (b, c) Effect of PS NPs and the endocytosis inhibitor 2-deoxy-D-glucose (2-DG) on the early endosomal marker EEA-1. (b) Immunoblot analysis of EEA-1 protein expression. Cells were pretreated with or without the 2-DG prior to exposure to different sizes (50, 100, 200 nm) and concentrations (0, 25, 50, 500 μg/mL) of PS NPs. (c) Corresponding quantitative analysis of EEA-1 protein and mRNA expression levels. Protein levels were normalized to β-actin, and all values were expressed relative to untreated controls (set as 1). The samples derive from the same experiment or parallel experiments and that gels/blots were processed in parallel. Data are represented as mean ± SD from three independent experiments.Statistical significance was determined by one-way ANOVA followed by Dunnett's post hoc test. **$p < 0.01$ compared to the PS NP-free group control. ##$p<0.01$, compared with the corresponding inhibitor-free group within the same treatment condition.

Figure S6. Quantitative analysis of autophagy-related markers protein expression in HaCaT

cells treated with PS NPs. Cells were exposed to 50 nm (a), 100 nm (b), or 200 nm (c) PS NPs at indicated concentrations (0, 25, 50, and 500 μg/mL) for 24 h. Protein levels of mTOR, LC3B, p62, Beclin-1, and ATG5 were determined by immunoblotting and normalized to β-actin. Bar graphs represent the relative protein expression (mean ± SD) from three independent experiments. Statistical significance was determined by one-way ANOVA followed by Dunnett’s post hoc test. *p < 0.05, **p < 0.01 compared with the control group (0 μg/mL) for each particle size.

Figure S1

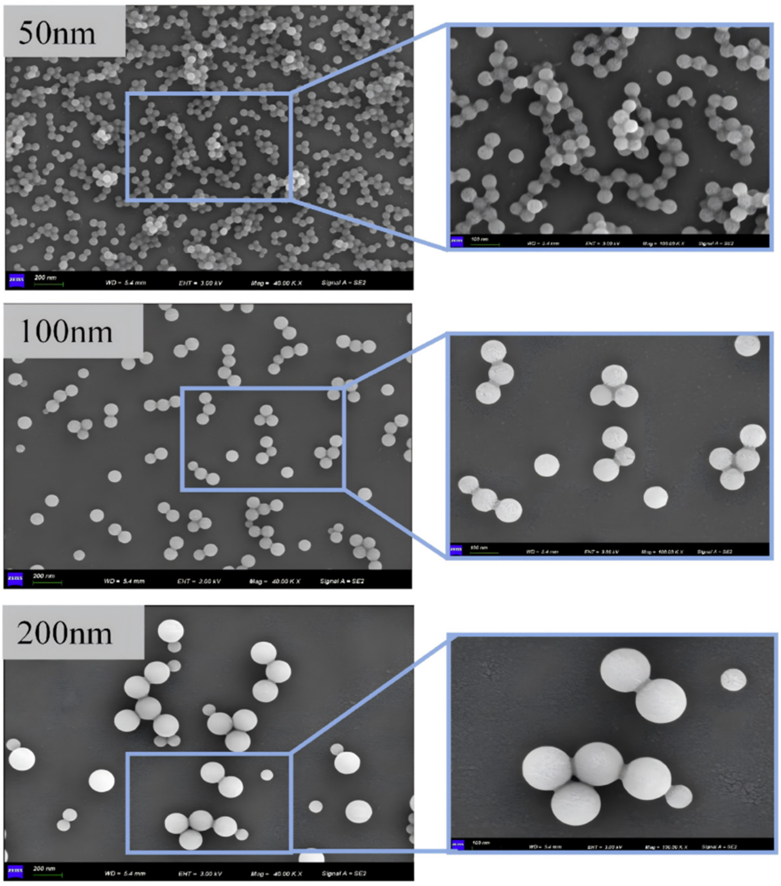

Figure S2

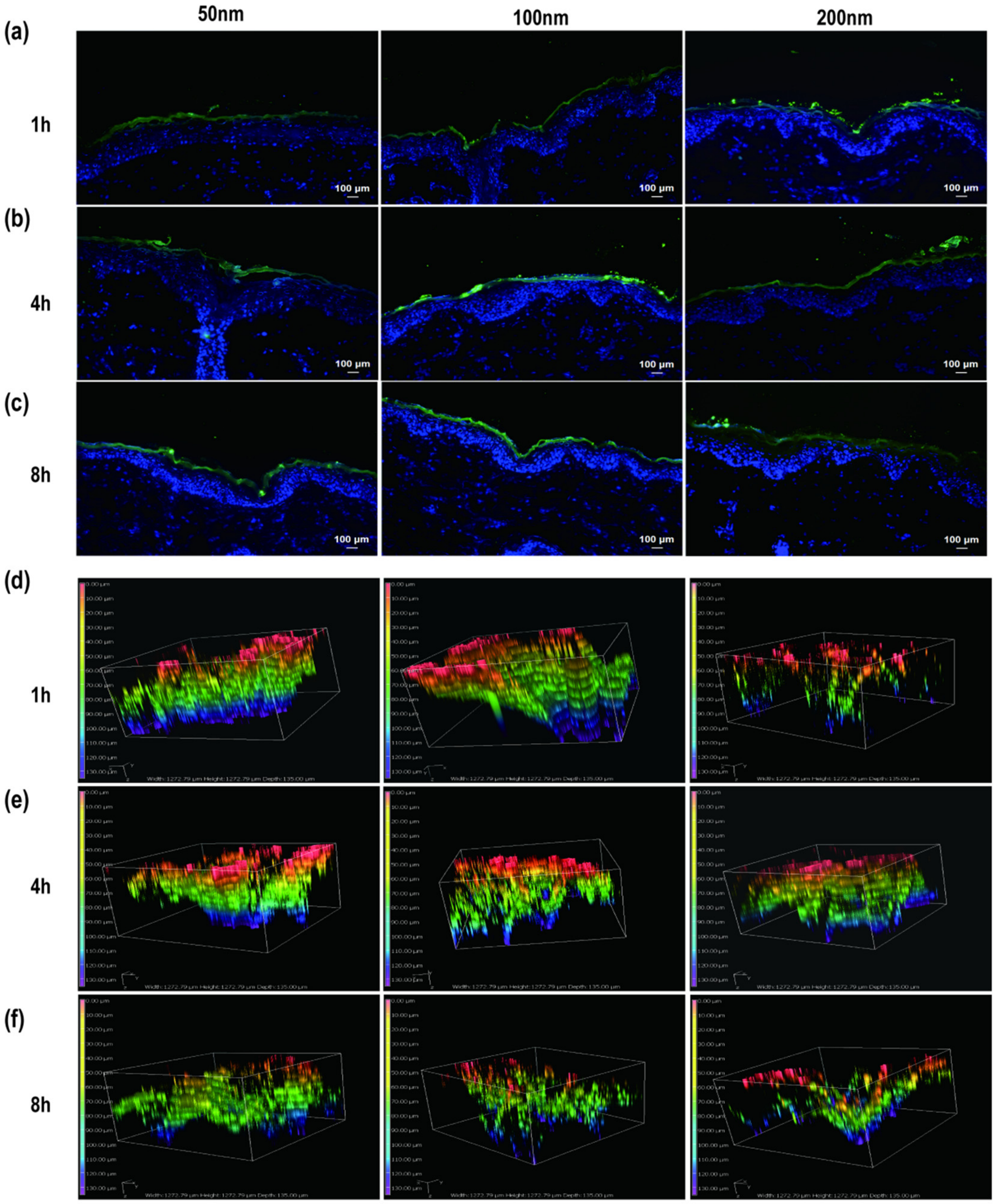

Figure S3

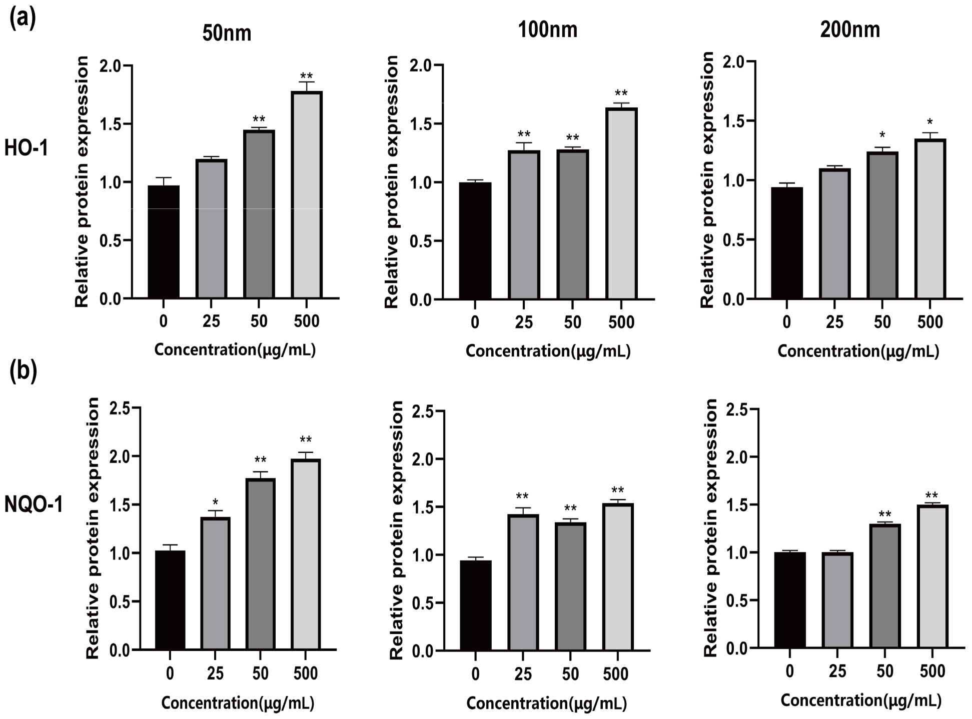
(a)
50nm
100nm
200nm
HO-1
Relative protein expression
2.0
1.5
1.0
0.5
0.0
0
25
50
500
Concentration(μg/mL)
*
**
(b)
NQO-1
2.5

Figure S4

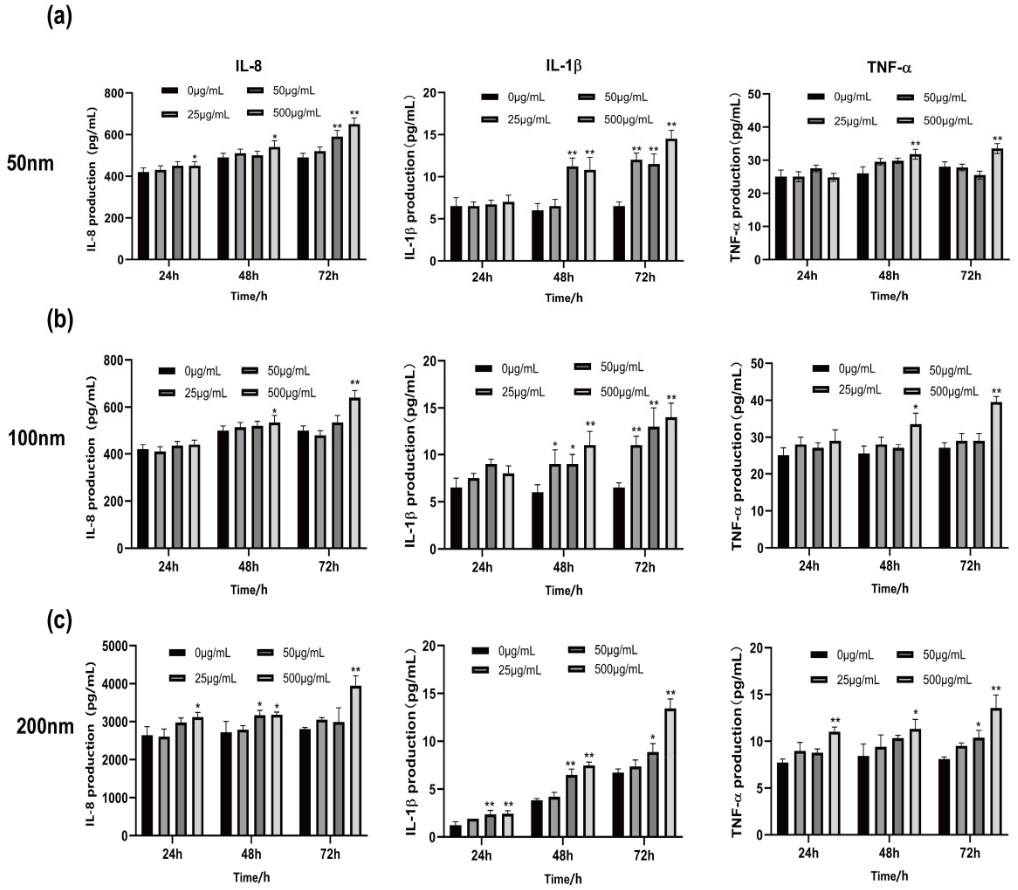

(a)
(b)
(c)
IL-8
IL-1β
TNF-α
50nm
100nm
200nm
0μg/mL
25μg/mL
50μg/mL
500μg/mL
IL-8 production (pg/mL)
IL-1β production (pg/mL)
TNF-α production (pg/mL)
24h
48h
72h
Time/h

Figure S5

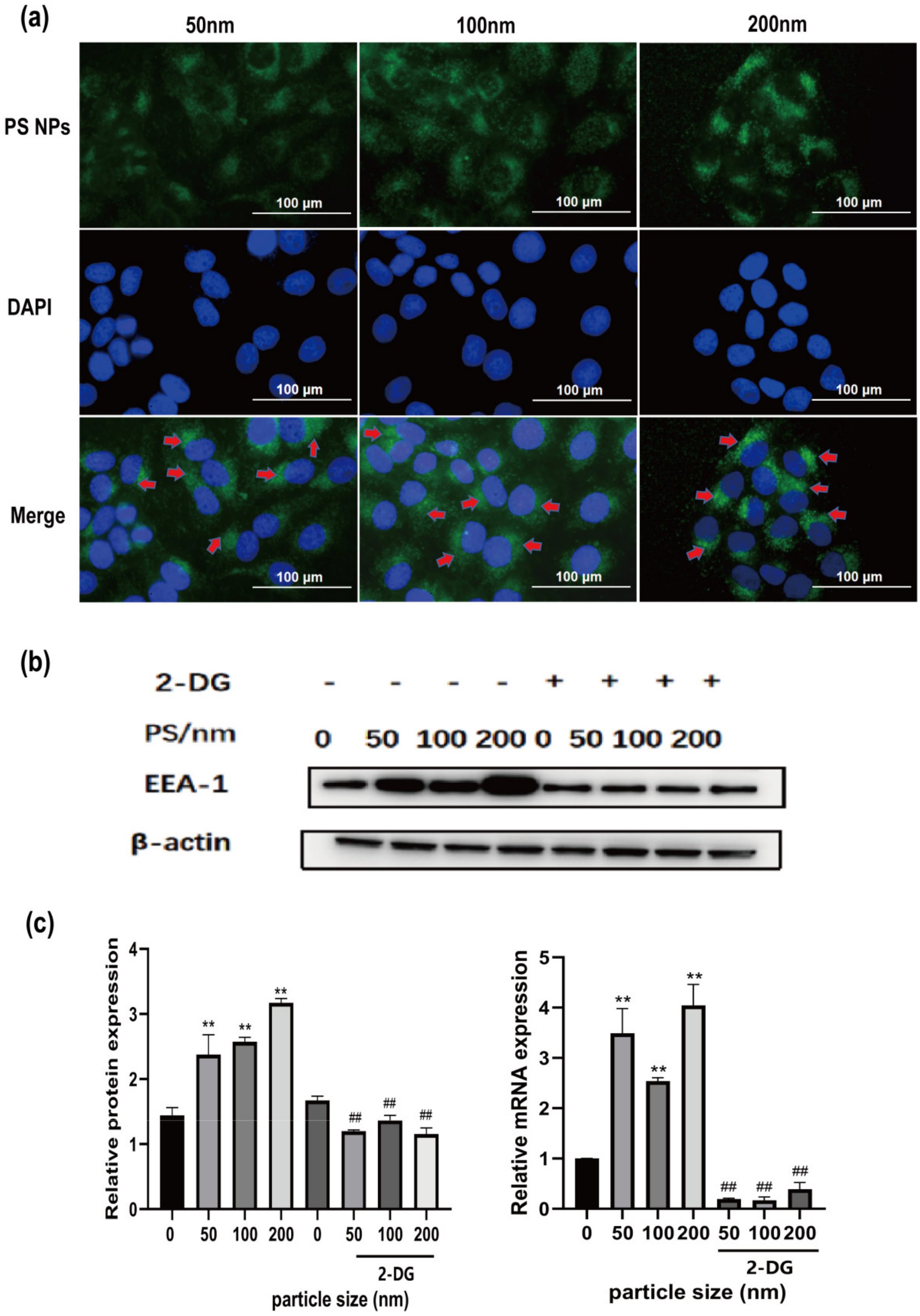

(a)
50nm
100nm
200nm
PS NPs
DAPI
Merge
100 μm
(b)
2-DG - - - - + + + +
PS/nm 0 50 100 200 0 50 100 200
EEA-1
β-actin
(c)
Relative protein expression
0 50 100 200 0 50 100 200
2-DG
particle size (nm)
Relative mRNA expression
0 50 100 200 50 100 200
2-DG
particle size (nm)

Figure S6

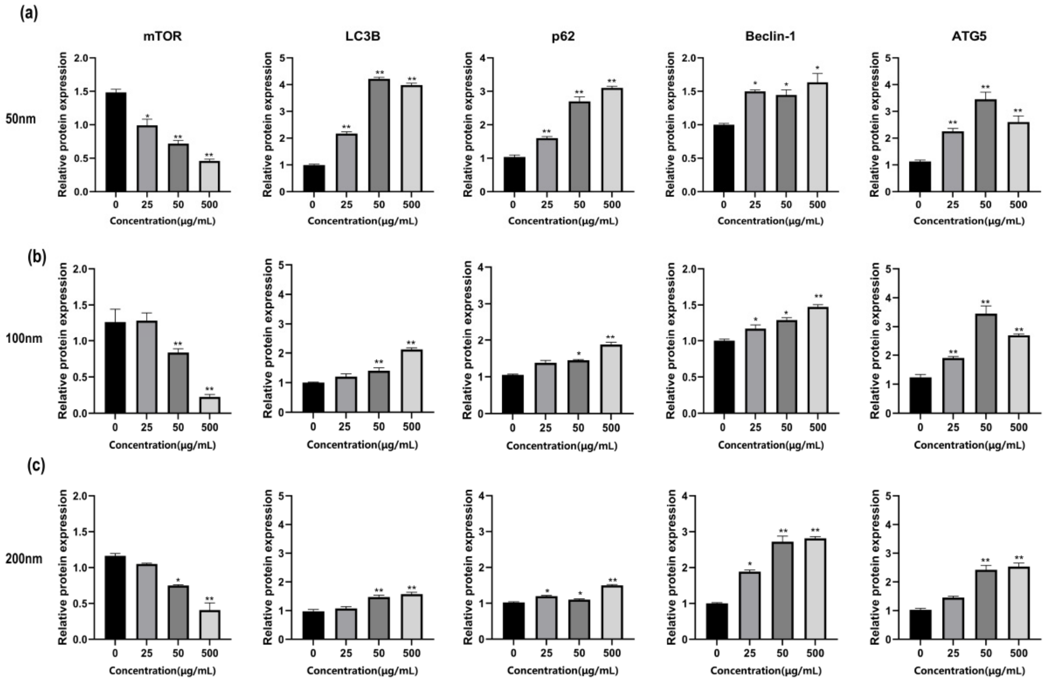
(a)
(b)
(c)
mTOR
LC3B
p62
Beclin-1
ATG5
50nm
100nm
200nm
Relative protein expression
Concentration(μg/mL)
